# Very large tunneling magnetoresistance in layered magnetic semiconductor CrI_3_

**DOI:** 10.1038/s41467-018-04953-8

**Published:** 2018-06-28

**Authors:** Zhe Wang, Ignacio Gutiérrez-Lezama, Nicolas Ubrig, Martin Kroner, Marco Gibertini, Takashi Taniguchi, Kenji Watanabe, Ataç Imamoğlu, Enrico Giannini, Alberto F. Morpurgo

**Affiliations:** 1 Department of Quantum Matter Physics, University of Geneva, 24 Quai Ernest Ansermet, CH-1211 Geneva, Switzerland; 2 Group of Applied Physics, University of Geneva, 24 Quai Ernest Ansermet, CH-1211 Geneva, Switzerland; 3 Institute of Quantum Electronics, ETH Zürich, CH-8093 Zürich, Switzerland; 4 National Institute for Materials Science, 1-1 Namiki, Tsukuba, 305-0044 Japan

## Abstract

Magnetic layered van der Waals crystals are an emerging class of materials giving access to new physical phenomena, as illustrated by the recent observation of 2D ferromagnetism in Cr_2_Ge_2_Te_6_ and CrI_3_. Of particular interest in semiconductors is the interplay between magnetism and transport, which has remained unexplored. Here we report magneto-transport measurements on exfoliated CrI_3_ crystals. We find that tunneling conduction in the direction perpendicular to the crystalline planes exhibits a magnetoresistance as large as 10,000%. The evolution of the magnetoresistance with magnetic field and temperature reveals that the phenomenon originates from multiple transitions to different magnetic states, whose possible microscopic nature is discussed on the basis of all existing experimental observations. This observed dependence of the conductance of a tunnel barrier on its magnetic state is a phenomenon that demonstrates the presence of a strong coupling between transport and magnetism in magnetic van der Waals semiconductors.

## Abstract (web summary)

Layered van der Waals compounds offer opportunities to visit new physical phenomena in two dimensional materials. Here the authors report large tunneling magnetoresistance through exfoliated CrI3 crystals and attribute its evolution to the multiple transitions to different magnetic states.

## Introduction

Investigations of layered van der Waals compounds are revealing a wealth of electronic phenomena, which can be controlled by varying the material thickness at the atomic scale^1–4^. Among these compounds, magnetic van der Waals semiconductors^5–19^ have remained virtually unexplored. These materials possess a unique potential for new physical phenomena, because magnetism occurs spontaneously without the need to introduce magnetic dopants as done in conventional magnetic semiconductors^20–22^, allowing—at least in principle—perfect crystalline order to be preserved. Indeed, the potential of magnetic van der Waals semiconductors has been made apparent by very recent experiments showing the occurrence of 2D ferromagnetism in atomically thin layers of Cr_2_Ge_2_Te_6_^16^ and CrI_3_^17^. So far however, essentially no experiment has been done to probe the transport and opto-electronic properties of these materials, and it remains to be determined whether their behavior deviates from that of conventional semiconductors, i.e., whether magnetism causes new interesting physical phenomena to appear. This can be expected because ab initio calculations predict the valence and conduction band of several ferromagnetic van der Waals semiconductors to be fully spin polarized^15,23–26^, implying a very strong coupling between the magnetic state and other electronic properties. Here we investigate experimentally these issues by performing transport and optical measurements on nano-fabricated devices based on exfoliated CrI_3_ crystals. In all devices investigated we find a very large tunneling magnetoresistance—as large as 10,000%—originating from abrupt transitions between different magnetic states of CrI_3_ that shows directly how the transport and magnetic properties are strongly coupled.

## Results

### Semiconducting characteristics of CrI_3_ devices

**Fig. 1:**
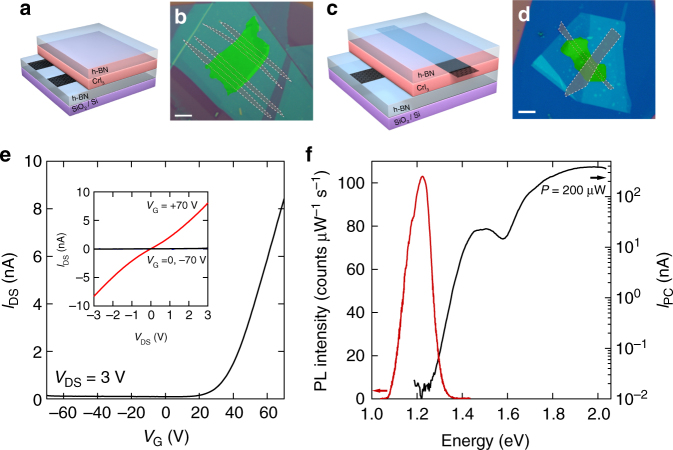
Semiconducting characteristics of CrI_3_. Scheme (**a**) and false-color optical micrograph (**b**) of a CrI_3_ field-effect transistor realized using few-layer graphene contacts, encapsulated between hexagonal boron nitride (hBN) crystals. The highly doped Silicon substrate covered by a 285 nm SiO_2_ layer is used as gate (the scale bar in **b** is 5 µm long). Scheme (**c**) and false-color optical micrograph (**d**) of a heterostructure consisting of bottom and top multilayer graphene contacts attached to an exfoliated CrI_3_ crystal ~7 nm thick (the entire structure is encapsulated between hBN crystals; the scale bar in **d** is 5 µm long). **e** Transfer characteristics of the field-effect transistor shown in **b** measured at room temperature with *V*_DS_ = 3 V applied between the two multilayer graphene contacts. The transistor turns on for positive gate voltage indicating electron conduction. The inset shows the source-drain current flowing between the graphene contacts as a function of *V*_DS_, for three values of gate voltage (*V*_G_ = −70 V, 0 V, and +70 V). **f** Dependence of the zero-bias photocurrent (black solid line) and photoluminescence (PL) intensity (red solid line) on the photon excitation energy (data taken at *T* = 4 K, on a device analogous to that shown in **d** with CrI_3_ of ~10 nm thick)

Past studies^5,6,10^ have shown that CrI_3_ exhibits a transition to an anisotropic ferromagnetic state with easy axis perpendicular to the layers (Curie temperature *T*_c_ = 61 K), accompanied by a singular behavior of the magnetic susceptibility below *T* ~ 50 K suggestive of a transition to a more complex magnetic state that remains to be understood (Supplementary Note 2). In contrast to the magnetic properties, virtually nothing is known about the opto-electronic response of this material and—to start exploring it—we have fabricated and investigated different types of devices (Fig. 1a–d, see Methods section and Supplementary Note 3 for details of the fabrication process). Figure 1a, b shows the schematics and an optical microscope image of a structure with graphene contacts attached to the bottom of an exfoliated CrI_3_ crystal, which we realized to implement a field-effect transistor (the doped Si substrate acts as gate). The observed gate and bias dependence of the current are shown in Fig. 1e and its inset: they conform to the expected transistor behavior and indicate that transport in CrI_3_ is mediated by electrons in the conduction band (since the transistor turns on at positive gate voltages). Figure 1c, d shows a second type of devices with contacts connected on opposite sides of an exfoliated thin CrI_3_ crystal, enabling photocurrent measurements. The photocurrent sets in sharply when the photon energy exceeds 1.2 eV (Fig. 1f), corresponding to the CrI_3_ band-gap^5,6^. This value is consistent with that inferred from the CrI_3_ photoluminescence spectrum that peaks at the same energy (see red line in Fig. 1f). Notably, the magnitude of the photocurrent is comparable to that measured on analogous devices based on crystals of more established van der Waals semiconductors, such as WS_2_^27^ or WSe_2_^28^. In contrast to the field-effect transistors, whose resistance was found in all cases to become unmeasurably high below 100 K, the photocurrent in vertical junctions persists down to low temperature. This suggests that the measurement of vertical transport in the direction perpendicular to the layers is possible at low temperature, and may be used to probe phenomena of magnetic origin.

### Tunneling transport in vertical junctions

**Fig. 2:**
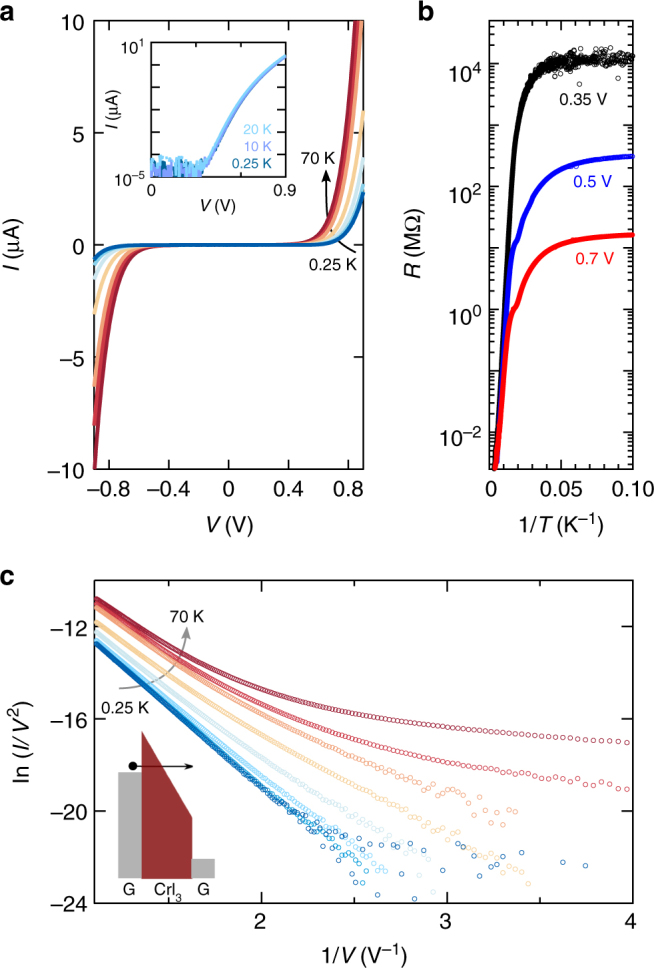
Electron tunneling in few-layer CrI_3_ vertical junctions. **a** Current measured on the device shown in Fig. 1d as a function of bias applied between the graphene contacts for *T* ranging from 0.25 to 70 K (the intermediate temperatures are 10, 20, 30, 40, 50, and 60 K). Below *T* = 20 K, the *I*–*V* curves become temperature independent as shown in the inset, indicating that transport is determined by tunneling (the overlapping area of the graphene contacts is 4 µm^2^ and the thickness of the CrI_3_ layer is ~7 nm). **b** Arrhenius plot of the resistance measured at different bias voltages (0.35, 0.5, and 0.7 V). **c** In the tunneling regime (i.e., for *T* < 20 K), ln(*I*/*V*^2^) is linearly proportional to 1/*V*, as expected for Fowler-Nordheim tunneling (charge carriers tunnel into the conduction band through band-gap of CrI_3_ that is tilted by the applied bias forming a triangular barrier, as illustrated schematically in the inset). The different curves correspond to measurements performed at the same temperatures as in **a**

We investigated vertical transport from room temperature down to *T* = 0.25 K, by measuring the *I*–*V* curves of devices such as the one in Fig. 1d. Representative data from one of these devices (Fig. 2a, thickness of CrI_3_ is ~7 nm, corresponding to ~10 monolayers) show strongly nonlinear *I*–*V* curves that are temperature independent for *T* < 20 K, whereas for larger *T* the current *I* at any given bias *V* increases with increasing temperature. The temperature evolution of the resistance *R* ( = *V*/*I*) measured at three different biases (*V* = 0.35 V, 0.5 V, and 0.7 V) is summarized in Fig. 2b: starting from room temperature, the resistance first increases in a thermally activated way down to *T* ~ 70 K (the typical value of activation energy found in different devices is *E*_a_ ~0.15 eV), where it starts to level off, and eventually saturates becoming temperature independent for *T* < 20 K.

The observed temperature independence indicates that for *T* < 20 K vertical transport is due to tunneling. Indeed, Fig. 2c shows that, for *T* < 20 K, ln(*I*/*V*^2^) scales proportionally to 1/*V*, the trend expected in the Fowler-Nordheim (FN) tunneling regime^29,30^. This regime occurs when the electric field generated by the applied voltage tilts the bands in the semiconductor, allowing carriers to tunnel from the electrode into the material^31^ (see the inset of Fig. 2c). Increasing the electric field effectively decreases the barrier thickness and causes an exponential increase of current. Theory predicts:

1 $${\rm ln}\frac{I}{{V^2}}\sim - \frac{{8\pi \sqrt {2m \ast } \phi _B^{3/2}d}}{{3{\rm hq}V}},$$

where *h* is Planck’s constant, *q* the electron charge, *d* the barrier thickness, m* the effective mass and *ϕ*_B_ the barrier height determined by the distance between the Fermi energy in the contact and the edge of the conduction band in CrI_3_ (the transistor measurements in Fig. 1e imply that electrons—and not holes—are responsible for the vertical tunneling current). If the effective mass is taken to be equal to the free electron mass—a plausible assumption in view of the rather narrow bands of CrI_3_^10,23,24^—we find that the barrier height is 0.25 eV, roughly comparable to the activation energy extracted from the measured temperature dependence of the resistance.

### Large tunneling magnetoresistance in vertical junctions

**Fig. 3:**
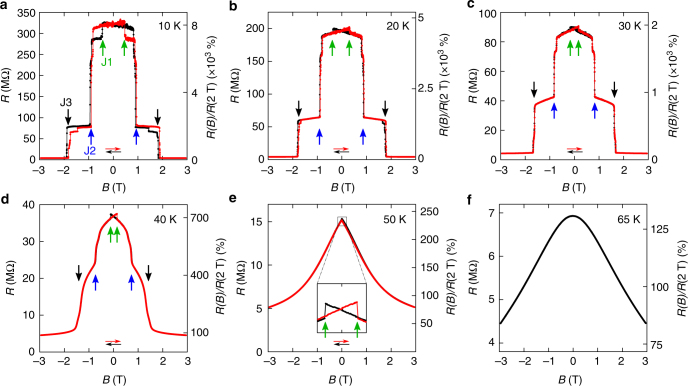
Large tunneling magnetoresistance in vertical junctions. **a**–**f** Tunneling resistance (left axis) and resistance ratio *R*(*B*)/*R*(2 T) (right axis) of the device shown in Fig. 1d, measured at the temperature indicated in each panel (with *V* = 0.5 V and **B** applied perpendicular to the CrI_3_ layers). The red and black dots correspond to data measured upon sweeping the field in opposite directions as indicated by the horizontal arrows of the corresponding color. The resistance ratio increases upon lowering temperature and reaches 8,000% at 10 K. The arrows of different color point to the magnetoresistance jumps that are seen in all devices, irrespective of the thickness of the CrI_3_ crystal. Jump J1 is always accompanied by hysteresis; at low temperature, jumps J2 and J3 occur in all devices at the same value of the applied magnetic field, irrespective of sweeping direction. All jumps shift to lower field values upon increasing temperature, and disappear above 50 K

Having established the mechanism of vertical transport and seen that measurements can be done well below the Curie temperature, we look at the effect of an applied magnetic field. The magnetoresistance measured at different temperatures between 10 and 65 K with the magnetic field applied perpendicular to the plane of CrI_3_ is shown in Fig. 3a–f (extra data are discussed in Supplementary Notes 4 and 5). Extremely large jumps are observed at low temperature, resulting in a total magnitude change up to 10,000% as **B** is increased from 0 to just above 2 T (Fig. 3a). The jumps pointed by the vertical arrows (J1, J2, and J3) are seen in all four measured devices. Jumps J2 and J3 occur at the same values of **B** irrespective of the CrI_3_ thickness, which in our experiments ranged from 5.5 to 14 nm (additional fine structure in the data depend on the specific device measured). Such a large magnetoresistance is striking as it is not commonly observed for electrons tunneling through non-magnetic materials. The sharp and well-defined values of applied magnetic field at which the resistance jumps are seen strongly suggest that the phenomenon originates from changes in the magnetic state of the CrI_3_ layers.

### Magnetic states of CrI_3_ and tunneling magnetoresistance

**Fig. 4:**
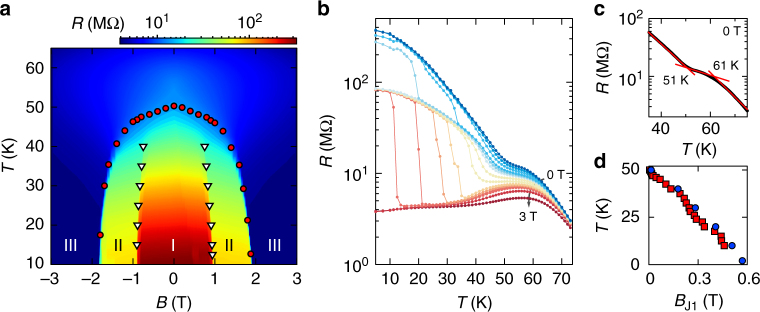
Temperature and magnetic field evolution of magnetic states in CrI_3_. **a** Color plot of the resistance of the device shown in Fig. 1d (in logarithmic scale), as a function of **B** and *T*. At low temperature, three clear plateaus signal the presence of different magnetic states (labeled I, II, III). The white triangles and red circles, obtained from the position of the resistance jumps as described in the text, outline the boundaries of these states, and show that the magnetoresistance features appear at *T* ≅ 51 K (i.e., in correspondence of the anomaly seen in the low-field magnetization; Supplementary Fig. 1c-d). **b** *T*-dependence of the resistance (in logarithmic scale) at different fixed values of *B*: three different values are attained at low-temperature, corresponding to the different magnetic states of CrI_3_. **c** *T*-dependence of the resistance measured at **B** = 0 T. The kink at *T* ≅ 51 K originates from the evolution of the boundaries between II and III; the ferromagnetic transition manifests itself as a kink around 61 K. **d** Temperature dependence of the position of jump J1 (clearly seen in Fig. 3 in linear scale; the logarithmic scale in Fig. 4a makes jump J1 difficult to discern). The blue and red symbols—measured 8 months after each other—demonstrate the excellent reproducibility and stability of encapsulated CrI_3_ devices

Identifying the nature of the magnetic states responsible for the tunneling magnetoresistance, and checking consistency with the known magnetic properties of bulk CrI_3_ is subtle. We start addressing these issues by investigating how the magnetoresistance depends on temperature. Upon increasing *T*, the resistance jumps shift position (compare Fig. 3a, c), with J2 and J3 becoming less sharp (Fig. 3d), and all features eventually disappear around 50 K, well below the Curie temperature of CrI_3_ (*T*_c_ = 61 K) at which the magnetization of the material appears. More detailed information is obtained by looking at the dependence of the magnetoresistance on both temperature and magnetic field. The color plot in Fig. 4a clearly shows that the resistance jumps define three states (that we label as I, II, and III). For *T* lower than ~40 K the states are separated by clear boundaries in the **B**-*T* plane, and well-defined transitions are seen irrespective of whether the boundary is crossed by varying **B** at fixed *T* or by changing *T* at fixed **B** (as shown in Fig. 4b), as expected for veritable phase transitions. This confirms that different magnetic states in CrI_3_ are responsible for the observed magnetoresistance.

Determining the precise onset temperature for the occurrence of tunneling magnetoresistance is also instructive. For *T* > 40 K the jumps are rounded into kinks whose position can be determined as shown in Fig. 4c. By following their evolution in the *B*- and *T*-plane we find that jump J3 (see the red circles in Fig. 4a) and J1 (see Fig. 4d, the feature associated to the small field hysteretic behavior visible in Fig. 3) start at the same temperature, *T* ≅ 51 K (rounding prevents the precise evolution of jump J2 to be followed above 40 K). Notably, 51 K corresponds exactly to the temperature of the singular behavior observed in the magnetic susceptibility of bulk crystals (Supplementary Fig. 1), indicative of a transition to a complex magnetic state different from a simple ferromagnet. This quantitative agreement therefore suggests that one of the states responsible for the large tunneling magnetoresistance observed in the experiments is the same state that manifests itself in the magnetic properties of bulk CrI_3_. The relation between the properties of bulk crystals and magnetoresistance is however more complex, as shown by magneto-optical Kerr effect (MOKE) measurements.

**Fig. 5:**
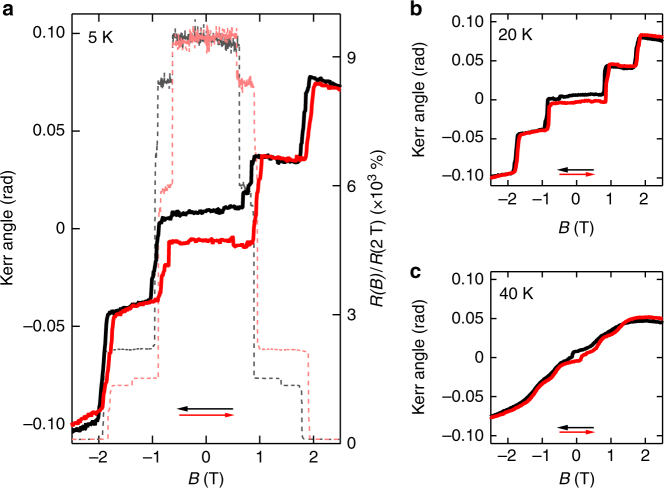
Magneto-optical Kerr effect in few-layer CrI_3_. **a** Comparison between the Kerr angle (solid lines, left axis) and the magnetoresistance (dashed lines; data plotted as resistance ratio *R*(*B*)/*R*(2T), right axis) measured on a same device at 5 K. Kerr angle is measured in Faraday geometry with magnetic field applied perpendicular to the plane of CrI_3_. Black and red curves correspond to sweeping the magnetic field in the direction pointed by the arrows of the corresponding color. The Kerr angle exhibits jumps at magnetic field values that coincide perfectly with the jumps observed in the magnetoresistance. **b**, **c** Kerr angle measured at 20 K and 40 K, respectively, as a function of magnetic field. The evolution with temperature is virtually identical to that observed for the magnetoresistance (Fig. 3), with features shifting to lower fields and becoming broader as temperature is increased

MOKE measurements exhibit a behavior analogous to that observed in transport, with sharp jumps in Kerr angle that are seen upon the application of a magnetic field perpendicular to the plane of CrI_3_ layers (Faraday geometry;^32^ see Fig. 5a). The jumps occur precisely at the same **B**-values at which the J2 and J3 magnetoresistance jumps are found. At *T* = 5 K a well-developed hysteresis in the magnetoresistance measurements is observed upon sweeping **B** up and down, and the same hysteresis is seen in the measurements of Kerr angle. The evolution of the **B**-dependence of the Kerr angle upon increasing *T* (Fig. 5b, c) also resembles what is observed in the magnetoresistance, with the jumps in the two quantities shifting and smearing in a very similar way. All the trends that we observed are qualitatively identical to the one reported earlier in atomically thin layers^17^, which—after submission of this manuscript—have also been reported to exhibit a tunneling magnetoresistance virtually identical to the one reported here^33,34^.

In thin atomic layers, MOKE measurements have been interpreted in terms of individual crystalline planes of CrI_3_, antiferromagnetically coupled at **B** = 0, switching to a ferromagnetic ordering upon application of an external magnetic field. Switching from antiferro- to ferromagnetic coupling may account for the occurrence of sharp jumps in the tunneling magnetoresistance, but is at odds with bulk magnetic properties. Indeed, if transitions from antiferromagnetic to ferromagnetic ordering of CrI_3_ layers would occur in crystals of all thicknesses, very large jumps should be observed in the bulk magnetization. Direct measurements (Supplementary Fig. 1) however show a near complete saturation of bulk magnetization occurring already at **B** ~ 0.3 T, and no change at **B** ~ 0.9 T and ~1.8 T in correspondence of the magnetoresistance jumps.

We conclude that the comparison of different experiments (*T-* and **B***-*dependence of the resistance, magnetic susceptibility, MOKE) indicate that the tunneling magnetoresistance originates from transitions in the magnetic state of CrI_3_. As for the details of the magnetic states involved, however, no simple interpretation straightforwardly reconciles all observations made on both bulk crystals and thin exfoliated layers. More work is needed to clarify this issue and two potentially important points are worth mentioning. One is that the evidence for the possible antiferromagnetic coupling proposed for exfoliated layers is inferred from the MOKE measurements, under the assumption of a direct relation between Kerr angle and total magnetization. However, it is known that in semiconducting systems a finite Kerr effect can be observed in the absence of a net magnetization as long as time reversal and inversion symmetry are broken^35,36^. Hence, transitions in the magnetic state without any change in magnetization (see illustrative examples in Supplementary Fig. 8 and discussion in Supplementary Note 6) could—at least in principle—cause jumps in Kerr rotation. This implies that to establish conclusively that an antiferromagnetic coupling is present it is very important to measure the magnetization of atomically thin CrI_3_ layers directly without simply relying on MOKE.

A second point to be made is that the jumps observed in MOKE and magnetoresistance could indeed originate from an antiferromagnetic coupling of adjacent CrI_3_ layers, but that interlayer antiferromagnetism only occurs in an interfacial region close to the crystal surface. If sufficiently thin, such an interfacial region would not be detected in bulk magnetization measurements, explaining why no magnetization jump is observed at 0.9 T and 1.8 T. Our observations that magnetoresistance occurs always at the same magnetic field values irrespective of applied bias, polarity, and thickness of the CrI_3_ flake up to 20 monolayers indicate that if an interfacial region is invoked, this region is rather thick. We estimate that crystals between 10 and 20 monolayers thick are still fully antiferromagnetically coupled, and understanding why such thick crystals exhibit a behavior that is very different from the one observed in the bulk is not obvious. One possibility is that the crystal structure of exfoliated layers in the surface region is different from that of bulk crystals. Interestingly, ab initio calculations show that if individual layers are stacked according to the high-temperature crystalline phase of CrI_3_, an antiferromagnetic interlayer coupling is energetically favorable as compared to the ferromagnetic coupling found in the low-temperature crystalline structure (see Supplementary Fig. 9 and discussion in Supplementary Note 7).

### Coupling between magnetic state and tunneling resistance

**Fig. 6:**
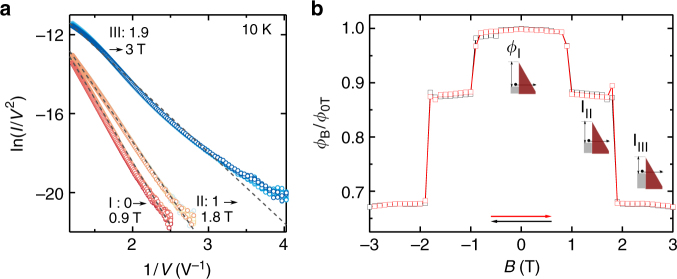
Coupling between magnetic state and tunneling resistance of CrI_3_ **a**, Plot of ln(*I*/*V*^2^) as a function of 1/*V* for **B** ranging between 0 and 3 T showing a nearly linear behavior with a **B**-dependent slope (*T* = 10 K). All data collapse on three different curves. **b** Magnetic field dependence of the barrier height extracted from the slopes of the curves in **a**, using Eq. (1)

Irrespective of the microscopic details of the underlying magnetic structure, finding that a step-like large modulation in the tunneling resistance of a magnetic insulator can be induced by a change in its magnetic state is a physical phenomenon that has not been reported earlier. It is therefore useful to look in detail at the *I–V* curves of our devices to see whether the experiments provide any indication as to the microscopic mechanism responsible for the change in tunneling resistance in the different magnetic states. This is done in Fig. 6a where ln(*I*/*V*^2^) is plotted versus 1/*V* for many different values of magnetic field. It is apparent that for different magnetic field intervals (I: 0–0.9 T; II: 1–1.8 T; III: 1.9–3 T) the *I–V* curves collapse on top of each other. In all cases the overall behavior is consistent with that expected from FN tunneling, but with a constant of proportionality between ln(*I*/*V*^2^) and 1/*V* (i.e., the slope of the three dashed lines in Fig. 6a) that is different in the three cases.

Microscopically, the constant of proportionality between ln(*I*/*V*^2^) and 1/*V* is determined by the transmission probability, i.e., to the extinction of the electron wavefunction tunneling through the CrI_3_ barrier. Within the simplest model of a uniform (i.e. layer independent) gap of CrI_3_, this quantity is determined by the electron effective mass and by the height of the tunnel barrier (Eq. (1)). Using Eq. (1) under the assumption that the effective mass remains unchanged, we can extract the height of the tunnel barrier from the slope of the ln(*I*/*V*^2^) versus 1/*V* relation. We find that—as shown in Fig. 6b—the barrier height is different in different magnetic states. The effect can be due to either a change in the band-gap or in the work-function of CrI_3_, consistently with ab initio calculations which shows that the occurrence of magnetism is accompanied by a modification in the material band structure^10,23,24^.

A similar conclusion—namely that different magnetic states have different transmission probability because of a difference in tunnel barrier height—holds true even if the change in tunneling magnetoresistance is due to a switch from an antiferromagnetic to a ferromagnetic interlayer ordering of the magnetization. The simplest model to describe this scenario consists in assuming that each layer has different barrier height for spin up and down^37,38^ (i.e., the height of the tunnel barrier is not spatially uniform). Calculations based on this model lead to *I–V* curves that also approximately satisfy Fowler-Nordheim behavior, with proportionality constant between ln(*I*/*V*^2^) versus 1/*V* that is different for a ferromagnetic or antiferromagnetic alignment of the magnetization in the individual CrI_3_ layers (Supplementary Fig. 10). This behavior is easy to understand qualitatively, because—despite not being spatially uniform—the average height of the tunnel barrier for the tunneling process that gives the dominant contribution to the current also depends on the magnetic state. Specifically, for ferromagnetic alignment, tunneling is dominated by the majority spin and the barrier height is the same—the smallest possible—in all layers. For antiferromagnetic coupling, electrons experience a barrier height that is alternating (depending on the layer) between the value expected for majority and minority spins, larger on average that the height experienced by the majority spins in the case of ferromagnetic alignment. These considerations imply that—at least at the simplest level—the analysis of the FN tunneling regime in the measured *I–V* curves cannot discriminate between different magnetic states. Nevertheless, they also do indicate that the magnetic state is coupled to the band structure, which is why the height of the tunnel barrier depends on the specific magnetic state.

## Discussion

The observation that the tunneling conductance through a CrI_3_ barrier depends strongly on the magnetic state of the material—a phenomenon that had not be observed previously in other systems—showcases the richness of physical phenomena hosted by van der Waals semiconductors. So far, these materials have attracted attention mainly for their opto-electronic and transport properties, but it is becoming apparent that their magneto-electronic response also exhibits fascinating and possibly unique properties. Inasmuch as CrI_3_ is concerned, future experiments should identify which other electronic phenomena, besides the tunneling conductance, are strongly affected by the magnetic state of the material. They should also aim at improving the quality of field-effect transistors (whose operation for CrI_3_ has been demonstrated here) to enable the investigation of gate-controlled transport at low temperature, in the magnetic state of the material. Obviously, however, experiments should be performed on a broader class of van der Waals magnetic systems, starting with those for which past measurements of the bulk magnetic response indicate the occurrence of transitions between states that can be controlled by the application of an experimentally reachable magnetic field.

After the submission of our manuscript different preprints have appeared on the cond-mat archive reporting observations closely related to the ones discussed here (refs. ^33,34,39^).

## Methods

### Crystal growth

High-quality crystals of CrI_3_ have been grown by the chemical vapor transport method in a horizontal gradient tubular furnace. To avoid degradation of the precursors and synthesized crystals the 1:3 mixture of Cr and I was sealed in a quartz tube (later placed in the furnace) under inert conditions (Supplementary Note 1).

### Sample fabrication

Multilayer graphene, h-BN (10–30 nm) and few-layer CrI_3_ flakes were exfoliated in a nitrogen gas filled glove box with a < 0.5 ppm concentration of oxygen and water to avoid degradation of the few-layer CrI_3_ crystals, which are very sensitive to ambient conditions (Supplementary Note 3). The heterostructures were then assembled in the same glove box with a conventional pick-up and release technique based on either PPC/PDMS or PC/PDMS polymer stacks placed on glass slides. Once encapsulated, the multilayer graphene electrodes were contacted electrically by etching the heterostructures by means of reactive ion etching (in a plasma of a CF_4_/O_2_ mixture) followed by evaporation of a 10 nm/50 nm Cr/Au thin film.

### Transport measurements

Transport measurements were performed either in a Heliox ^3^He insert system (Oxford Instruments, base temperature of 0.25 K) equipped with a 14 T superconducting magnet, or in the variable temperature insert of a cryofree Teslatron cryostat (Oxford Instruments, base temperature of 1.5 K) equipped with a 12 T superconducting magnet. The latter system is also equipped with a sample rotator making it possible to align the sample so that the magnetic field is either parallel or perpendicular to the CrI_3_ layers. The *I*–*V* curves and magnetoresistance were measured with a Keithley 2400 source/measure unit and/or home-made low-noise voltage sources and current amplifiers.

### Optical measurements and MOKE

Photoluminescence measurements were performed in a home tailored confocal micro-photoluminescence setup in back-scattering geometry (i.e., collecting the emitted light with the same microscope used to couple the laser beam onto the device). The light collected from the sample was sent to a Czerny–Turner monochromator and detected with a liquid nitrogen cooled Si CCD-array (Andor emCCD). The sample was illuminated with the 647.1 nm laser line of an Ar–Kr laser at a power of 30 µW. The data were corrected to account for the nonlinear CCD response in this spectral region. The same setup was used for the photocurrent measurement but in this case the devices were illuminated using a Fianium supercontinuum laser coupled to a monochromator, providing a beam of tunable wavelength with spectral width of 2 nm and stabilized power.

The magneto-optical Kerr effect (MOKE) measurements were performed in a cryostat with a 12 T superconducting split-coil magnet. The sample was illuminated with linear polarized light at 632.8 nm and 50 µW provided by a power stabilized HeNe laser. The reflected beam was split using a polarizing beam splitter cube and the s- and p-components measured simultaneously with Si-photodiodes and lock-in detection. A linear contribution to the measured Kerr angle, which stems from Faraday rotation in the cold objective lens, has been measured independently and was subtracted from the data in order to obtain the traces shown in Fig. 4.

### Data availability

All relevant data are available from the corresponding authors on request.

## Electronic supplementary material

Supplementary Information
