# Supplementary material for: Very large tunneling magnetoresistance in layered magnetic semiconductor CrI3
Source: Nat Commun. 2018 Jun 28;9:2516. doi: 10.1038/s41467-018-04953-8 (PMC6023911; doi:10.1038/s41467-018-04953-8)
Supplement: Supplementary file 1 — Supplementary Information [file 41467_2018_4953_MOESM1_ESM.pdf]

Very Large Tunneling Magnetoresistance in Layered Magnetic Semiconductor CrI_3

Supplemental Information

Wang *et al*.

Supplementary Note 1. Crystal growth and elementary characterization of bulk crystals

Crystals of CrI_3 have been grown by the Chemical Vapor Transport method[1]. The elemental precursors Cr (lumps, 99.95% pure, Materials Research SA) and I (crystalline, 99.99+% pure, Alfa Aesar), mixed in the nominal ratio 1:3 with a total mass of 0.3 g, were inserted in a quartz tube inside a glove box filled with 99.9999% Ar. The quartz tube (inner diameter 8 mm) was then tightly connected to a pumping line and evacuated down to ~10^{-4} mbar, with intermediate Ar flushing. The tube was subsequently sealed to a length of ~10 cm and placed horizontally in a tubular furnace with the hot end at 720 ℃ and the cold end at ~ 640 ℃. The thermal treatment lasted 7 days, at the end of which the furnace was switched off and the samples cooled down to room temperature inside the furnace. Shiny, plate-like, dark greyish crystals were found to grow at the cold end of the tube and were easily extracted. Smaller, slightly darker and less shiny ones were also found to grow in the hot zone.

The crystals were characterized by X-ray diffraction in a powder diffractometer (Bragg-Brentano geometry, using a Cu-K X-ray source), which confirmed the C12/*m* crystal structure, and by electron dispersive X-ray spectroscopy (EDS) in a scanning electron microscope, which confirmed the 1:3 atomic ratio in the final crystals. No traces of CrI_2 were found, within the sensitivity of our XRD and EDX probes. Bulk crystals have been proved to remain stable in air for a time long enough for the structural and chemical characterization.

Supplementary Note 2. Magnetism in bulk CrI3

CrI_3 is a layered material (see Supplementary Figure 1a) known to exhibit a transition to an anisotropic ferromagnetic state (Curie temperature T_c =61 K), showing characteristics of an extremely soft ferromagnet, in which the magnetization due to the spins on the Cr atoms is oriented perpendicular to the layers[1-3] (as discussed in Ref 1, the ultra-soft behavior at low magnetic field is likely due to the formation of up and down domains, with domain walls that can move easily through single crystals). Such magnetic behavior is also observed in the magnetization measurements performed on bulk CrI_3 crystals grown in our laboratory (Supplementary Figure 1 b-d; see below for details regarding the measurements). In the temperature dependence of the magnetization an anomaly is clearly present at T ~ 51 K, i.e., well below the Curie temperature (see insets of Supplementary Figure 1c and d). This anomaly is likely due to a second magnetic transition to a state with a more complex spin configuration, in which the spins are not perfectly aligned in the direction perpendicular to the layers[1].

All magnetic measurements have been performed in a variable temperature MPMS3 SQUID magnetometer (Quantum Design). The horizontal rotator option was used to carefully align the *ab*-plane of CrI_3 crystal to be perpendicular or parallel to the applied magnetic field.

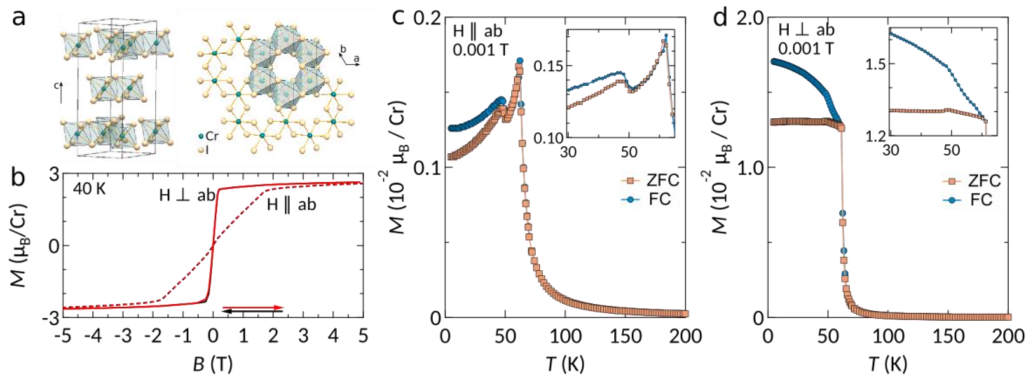

Supplementary Figure 1 | Magnetism in bulk CrI_3. a, Structure of CrI_3 crystal (the scheme represents low temperature equilibrium phase of CrI_3). Left: unit cell; right: top view of the *ab* plane. **b**, Anisotropic magnetic field dependence of the magnetization of bulk CrI_3 measured at 40 K (solid lines: **B** applied perpendicular to the CrI_3 layers; dashed lines: **B** applied parallel to the CrI_3 layers), showing the typical behavior of an extremely soft ferromagnet (i.e., the remnant magnetization at **B** = 0 T is vanishingly small). **c-d**, Zero field cooled (orange squares) and field cooled (blue circles) average magnetic moment per Cr atom measured with **B** = 1 mT applied parallel (**c**) or perpendicular (**d**) to *ab*-plane of CrI_3, enabling the determination of the Curie temperature, T_c = 61 K. The insets in figures (**c**) and (**d**) zoom-in on the region around 50 K where an anomaly suggestive of an additional phase transition is clearly seen.

Supplementary Note 3. Sensitivity of CrI_3 to atmospheric conditions and encapsulation

Exfoliated thin (<20 nm) CrI_3 crystals fully degrade within minutes upon exposure to air. The degradation leads to complete decomposition within 15 minutes, even for relatively thick flakes (i.e., much thicker than monolayers), as shown in Supplementary Figure 2 a-c. To avoid degradation, CrI_3 flakes were exfoliated in the inert atmosphere of a glove box (< 0.5 ppm of water and oxygen) and encapsulated in air-stable materials such as graphene or hexagonal boron nitride (hBN), using a dry transfer technique[4]. Supplementary Figure 2 d-f illustrates the situation with successive optical microscope images of different CrI_3 flakes on a large hBN crystals: only the CrI_3 flakes protected by a top graphene monolayer (black dotted line in Supplementary Figure 2) do not exhibit degradation. The other flakes strongly degrade within minutes.

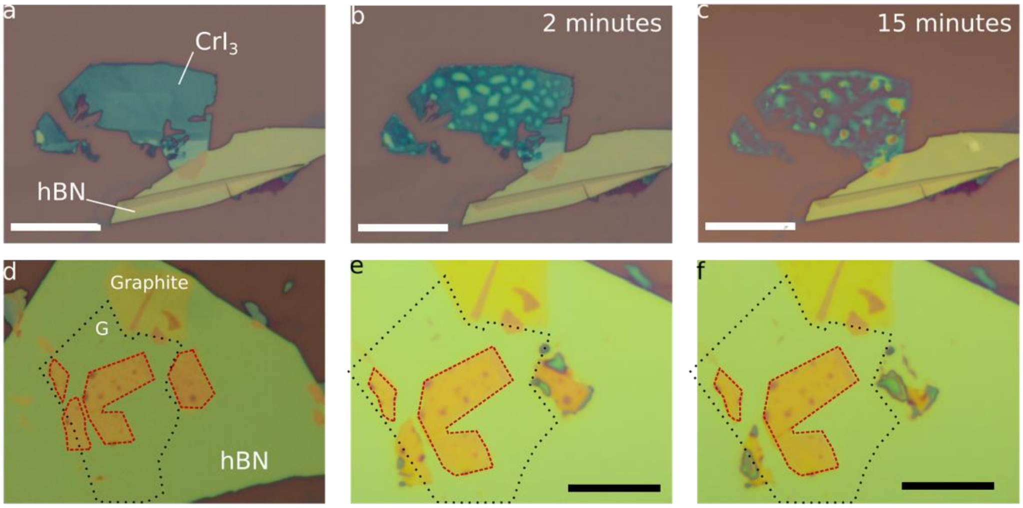

Supplementary Figure 2 | Degradation of CrI_3 thin flakes in air and protection by encapsulation. a-c Optical microscope images of bare CrI_3 thin flake as exfoliated (**a**), after 2 minutes of exposure to ambient (**b**) and after 15 minutes (**c**). The extremely fast degradation process of the material is clearly apparent. **d-f** Optical microscope images of encapsulated CrI_3 flakes (whose contour is indicated with the red dashed line) as prepared (**d**), after 2 minutes of exposure to air (**e**) and after 15 minutes (**f**). The images demonstrate that the CrI_3 flakes are effectively protected by the bottom hBN crystal and the top graphene monolayer (the contour of the top monolayer graphene used for encapsulation is indicated by the black dotted line). The scale bar in all images is 10 μm long.

In all devices realized to study transport properties (i.e., field effect transistors and vertical junctions), the structures consisting of CrI_3 and the multilayer graphene contacts were encapsulated with insulating hBN flakes. Optical microscope images of one device are shown in Supplementary Figure 3 a and b, just after the encapsulation and after metal contact deposition, respectively. No degradation was observed during and after the fabrication process, which included electron beam (e-beam) lithography, PMMA development, reactive ion etching (done to expose the graphene contacts far from the CrI_3 crystal, by etching away locally the top hBN layer), e-beam evaporation, and lift-off. Indeed, once properly encapsulated, the devices are stable virtually forever. As an indication, the blue squares and red dots shown in Fig. 6d of the main text were measured 8 months apart without any apparent change in the result. Also, the atomic force microscope image in Supplementary Figure 3c, taken after months of exposure to ambient conditions, shows no sign of degradation.

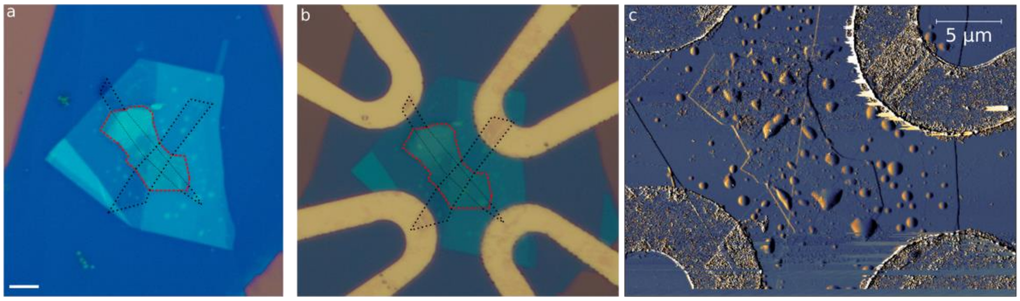

Supplementary Figure 3 | Stability of an encapsulated CrI_3 device. a Optical microscope image of a vertical Graphene-CrI_3-Graphene junction encapsulated between two hBN crystals as extracted from the glove box where the structure was assembeld. The scale bar is 5 µm long. **b** Optical image of the same heterostructure, taken after the multi-layer graphene was side contacted by metal. **c** Atomic force microscopy image of the same device, recorded after the transport measurements. The thickness of the CrI_3 flake was determined to be ~ 7 nm ("bubbles" are present in the structure but it is not possible to determine between which of the layers). These images demonstrate that encapsulation of CrI_3 is a very robust method against degradation in ambient conditions and withstands standard nano-fabrication techniques.

Supplementary Note 4. Reproducible magnetoresistance behavior

The key aspects of "vertical" magneto-transport behavior discussed in the main text have been observed in all the samples that we have investigated, as illustrated by the data taken on four different devices shown in Supplementary Figure 4 and 5. In particular, Supplementary Figure 4 and its inset show that jumps J1, J2, J3 (see main text) are present in all devices: jumps J2 and J3 occur at the same values of magnetic field in different devices, whereas jump J1 (see the inset of Supplementary Figure 4) is always accompanied by a hysteresis in the magnetoresistance. It is worth noting that in recent experiments on van der Waals heterostructure of CrI_3 and WSe_2, a switch in the optical response has been reported to occur at the exact same magnetic field values as the "jump" J2 and J3[5].

The magnitude of the MR depends on the applied bias (as shown in Supplementary Figure 5), and typically decreases upon increasing the applied voltage. For each device, the largest magnetoresistance was observed by applying the lowest possible voltage (the smallest voltage is limited by the sensitivity with which we can measure the current), and we found values ranging from 12'500% to 16'600%. In contrast to MR magnitude that was found to depend on bias, the values of the magnetic field at which the MR jumps J2 and J3 occur is the same for all values of applied voltage. Finally, the robust nature of the MR behavior is further illustrated by the temperature evolution of the magnetoresistance with bias voltage of 0.7 V shown in Supplementary Figure 6, which exhibits an identical behavior as the data taken at 0.5 V (shown in Supplementary Figure 3).

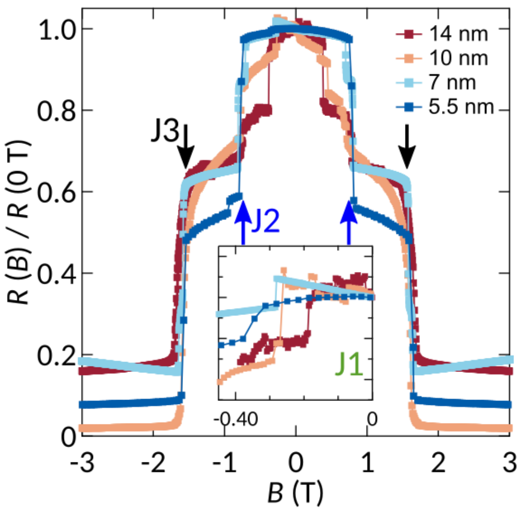

Supplementary Figure 4 | Magnetoresistance measured in different devices. Magnetic field dependence of the resistance normalized to zero field for four vertical junctions realized with exfoliated CrI_3 crystals of different thickness. The magnetic field is applied perpendicular to CrI_3 layers and swept from positive to negative polarity. The applied voltage bias is 3 V for 14 nm device, 1.3 V for 10 nm device, 0.7 V for 7nm device and 0.3 V for 5.5 nm device. The measurements are done at 30 K, a temperature at which very little hysteresis is seen for Jump J2 and J3. All the devices show the resistance jump J2 and J3 at the same field value, around +/- 0.8 T and +/- 1.5 T, for 30 K. The magnitude of the change in resistance at the jump depends on the applied bias (see Supplementary Figure 5), but the maximum values of magnetoresistance *R*(B)/*R*(2T) are comparable in different devices (12 500% for the 5.5 nm device, 16 600% for the 7 nm device and 15 000% for the 10 nm device; for the 14 nm device we have not systematically investigating the magnitude of the MR for different bias). The insert zooms in the data around zero field and demonstrates that the jump J1 is also always present in all devices.

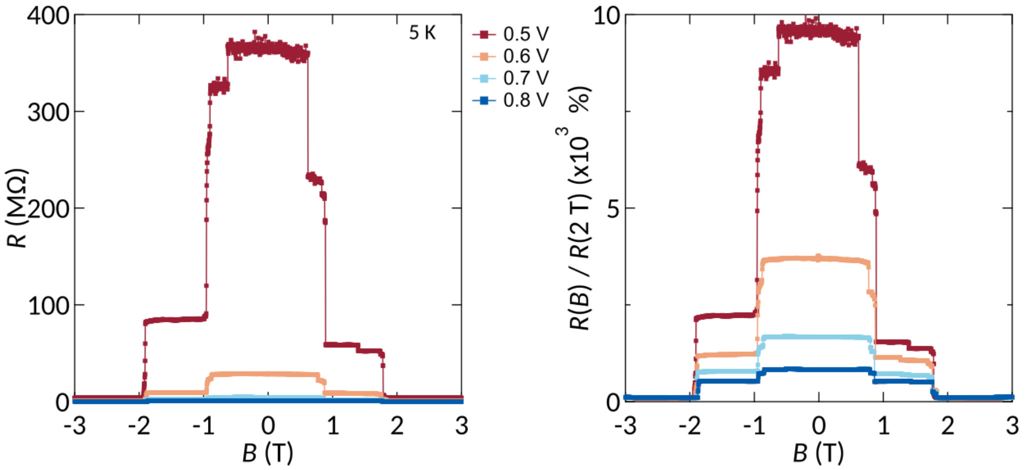

Supplementary Figure 5 | Magnetoresistance measured at different voltage bias. a-b, Magnetic field dependence of the resistance (**a**) and resistance ratio *R(B)/ R(*2T*)* (**b**) of the same device whose magneto-resistance is discussed in the main text, measured at 5 K and four different voltages biases (0.5 V, 0.6 V, 0.7 V and 0.8 V, as indicated in the legend). Despite the smaller resistance observed at higher applied bias, due to the reduction of the tunnel barrier width by the higher electric field, all the curves show the presence of the jumps J2 and J3 occurring at the same value of magnetic field.

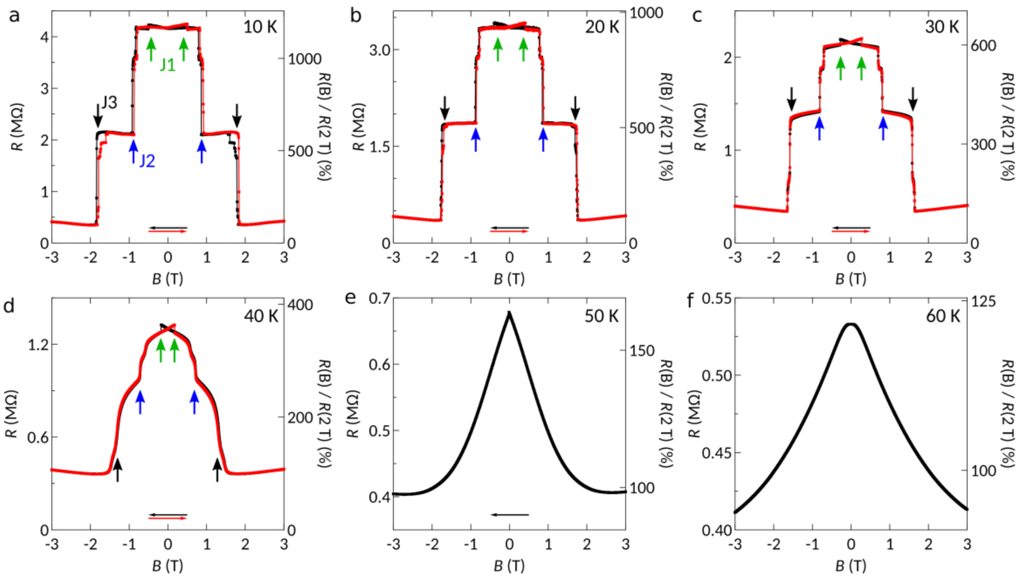

Supplementary Figure 6 | Temperature evolution of magnetoresistance at a different voltage bias. a-f Magnetoresistance measured with a voltage bias of 0.7 V at 10 K (**a**), 20 K (**b**), 30 K (**c**), 40 K (**d**), 50 K (**e**) and 60 K (**f**). The magnetic field is applied perpendicular to the CrI_3 layers. The black and red curves correspond to different sweep directions of the field, as indicated by the horizontal arrows.

Supplementary Note 5. Magnetoresistance due to an in-plane field

In the main text we only discussed the magnetoresistance observed upon applying a magnetic field perpendicular to the plane of CrI_3. However, a magnetic field applied parallel to the plane also leads to a large magnetoresistance. Supplementary Figure 7 shows the magnetoresistance with **B** applied parallel to the plane measured on a device different from the one discussed in the main text (the thickness of CrI_3 crystal in this device is 5.5 nm). The parallel magnetoresistance sets in at a similar temperature and has a comparable magnitude and sensitivity to the applied bias as the one measured when the field is applied perpendicular to the plane. The main differences are that 1) the magnetoresistance exhibits a continuous evolution without discrete steps; 2) the field needed to fully align the spins is somewhat larger than what needed when the field is applied perpendicular to the plane. The value of the resistance measured when the applied field is sufficiently large to align all the spins is approximately the same (although not identical) irrespective of whether the field is applied parallel or perpendicular to the CrI_3 layers. These measurements confirm the strongly anisotropic nature of magnetism in CrI_3.

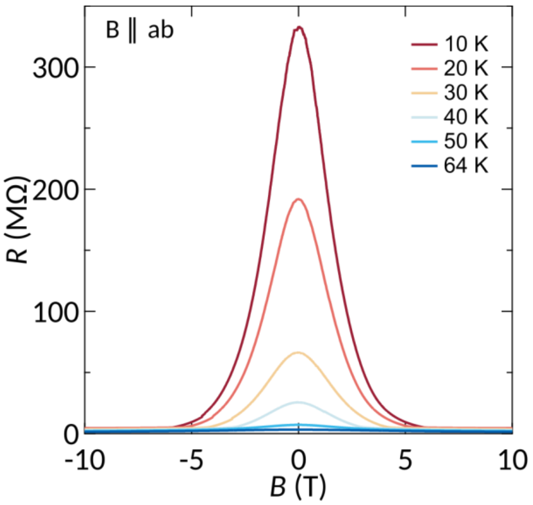

Supplementary Figure 7| Magnetoresistance with field parallel to the plane. Temperature evolution of magnetoresistance when the applied magnetic field is parallel to CrI_3 layers. In contrast with the case of a perpendicular applied magnetic field, where discrete jumps are observed, the resistance changes smoothly as function of field at all the measured temperatures.

Supplementary Note 6. Spin configurations with comparable magnetization.

In the main text we have shown that the magnetoresistance observed in our devices originates from the transition between different magnetic states. In the magnetic field range where the transitions occur, measurements of the bulk magnetization show only very small changes (less that approximately 5%) and no “jumps”. This implies that the different magnetic states responsible for the magnetoresistance jumps must have approximately the same magnetization. Supplementary Figure 8a-c are meant to illustrate with simple 1D schemes the qualitative aspects of spin configurations corresponding to different magnetic states having the same –or only minorly different– magnetization. To avoid misunderstandings: the purpose of this discussion is only to illustrate that the constraint that the magnetization does not changes (or changes very little) does not prevent transition between different magnetic states that can lead to different tunneling magnetoresistance values. In fact, since CrI_3 is formed by stacking together planes with the spins on the Cr atoms forming a 2D hexagonal lattice in each plane, a large variety of different magnetic states conceptually analogous to those shown in Supplementary Figure 8 can be conceived.

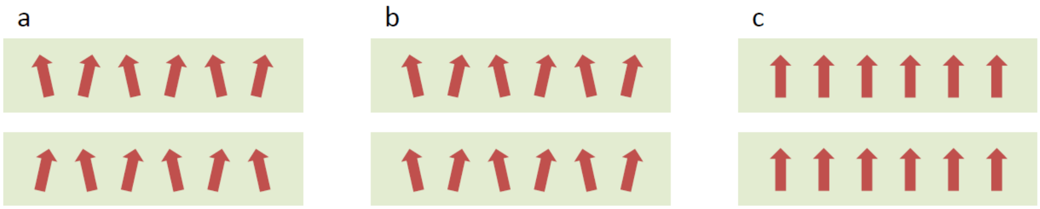

Supplementary Figure 8| Examples of different magnetic states with almost same magnetization. a-c spin configurations in two adjacent layers of CrI_3 (in a bulk crystal these two layers are repeated periodically in the direction perpendicular to the plane). The spins in (**a**) and (**b**) are almost fully polarized perpendicular to the plane, with a small canting due to a spin component exhibiting in-plane antiferromagnetic ordering. The only difference between the state in (**a**) and (**b**) is the spatial alignment of the spin in neighboring planes. The different alignment correspond to different magnetic states, without any different in magnetization. (c) corresponds to the high field spin configuration, with all the spin perfectly aligned perpendicularly to the planes.

Supplementary Note 7. Exchange coupling from first-principles in bilayer CrI_3 with different stacking order

In order to assess possible mechanisms to explain the presumed existence of inter-layer antiferromagnetic coupling in thin CrI_3 samples, we investigate here the magnetic ground state of CrI_3 bilayers as a function of the stacking order. Indeed, CrI_3 undergoes a structural phase transition from a high-temperature monoclinic phase (space group C2/m) to a low-temperature rhombohedral phase (space group R-3). The transition is not sharp and extends over a finite temperature range depending on the cooling history of the sample[1]. The main difference between the two phases consists mainly in the stacking

order of the layers, with the structure of the layers themselves remaining almost unaffected across the transition. In the high-temperature phase the layers are displaced along the zigzag direction by approximately a/3, while in the low-temperature phase there is a ABC stacking, with each layer displaced along the armchair direction by $a/\sqrt{3}$. It seems possible that in thin crystals, the phase transition might occur differently, or that the presence of multilayer graphene contacts might affect the transition, so that thin crystals or their outermost layers might display a different stacking order with the respect to the bulk R-3 phase.

To analyze the consequences of such a scenario, we have considered two layers (for simplicity) of CrI_3 and have investigated their electronic and magnetic properties for different stacking order using state-of-the-art first-principles density-functional-theory simulations. Starting from an AA stacking, with one layer exactly on top of the other, we have computed the energy of the ferromagnetic (FM) and antiferromagnetic (AFM) configurations for several horizontal displacements between the two layers. In Supplementary Figure 9a we show the FM energy as a function of the displacement (a similar pattern is observed also for AFM). The minimum energy is for AB (or AC) stacking, in agreement with the experimental observation in bulk CrI_3. Two additional local minima correspond to the high-temperature (HT) and AA stacking. All other minima are equivalent by symmetry to the previous ones. In Supplementary Figure 9b we instead show the energy of the FM (blue) and AFM (orange) configurations along a path that goes from AB to AA stacking, passing through the HT displacement (see also the white solid line in Supplementary Figure 9a). In agreement with previous calculations for bulk CrI_3, with AB stacking the FM configuration is the most stable. One would thus expect free-standing few-layers to display AB(C) stacking and ferromagnetic inter-layer coupling according to DFT simulations. If one allows the layers to have a stacking similar to the high-temperature phase, we instead have an almost perfect degeneracy between FM and AFM configurations, with a mild preference for the latter that might be further enhanced by dipolar interactions. This suggests that if some or all the layers of thin samples are frozen for some reason in the HT stacking, e.g. because of the interaction with the encapsulating layers, we could have AFM interlayer coupling. These considerations, therefore, could explain why thin layers exhibit magnetic properties characteristic of an antiferromagnet (under the assumption that the Kerr effect measurements effectively probe their magnetization) whereas the bulk magnetization exhibits ferromagnetism.

Calculations have been performed using Quantum ESPRESSO[6] with spin-polarized van-der-Waals-compliant functionals[7]. An energy cutoff of 60 Ry for wave functions and 480 Ry for the density have been used, while the Brillouin zone was sampled using a 6x6x1 Monkhorst-Pack grid. Spurious Coulomb interactions between artificial periodic replicas of the bilayers have been removed using a real-space cut-off[8].

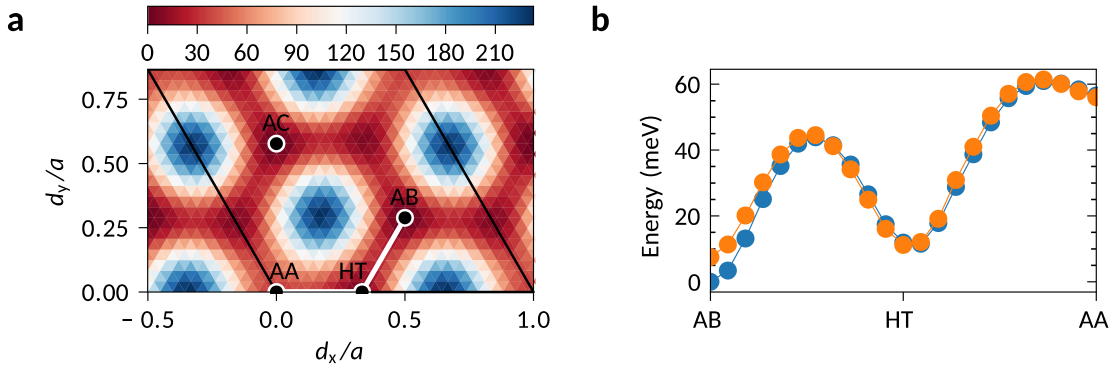

Supplementary Figure 9| Influence of stacking order on the magnetic state of bilayer CrI_3. a. Colorplot of the energy of bilayer CrI_3 in the ferromagnetic configuration as a function of the horizontal displacement *d* between the layers along the x and y directions in units of the lattice constant *a*. A similar behavior is found also for an antiferromagnetic configuration. The colorbar is the energy difference in meV with respect to the lowest energy AB stacking. The black solid line highlights the primitive cell. **b.** Energy of the ferromagnetic (blue) and antiferromagnetic (orange) configurations as a function of the displacement between the two layers in CrI_3 along a path going from the lowest-energy AB stacking to AA staking, passing through the high-temperature (HT) order (see also the white solid line in panel **a**).

Supplementary Note 8. Tunneling current in the presence of a non-uniform and spin-dependent potential barrier

In the main text, we have shown that the measured current through CrI_3 is due to tunneling at low temperatures and follows the Fowler-Nordheim (FN) law reported in Eq. (1). The FN behavior survives also in the presence of a vertical magnetic field, although the current changes in a step-like fashion as a function of the applied B-field, giving rise to the large magnetoresistance discussed in the main text. We have argued that this effect can be accounted for by a variation in the effective barrier height entering the FN expression for the tunneling current (see Fig. 6) that is different in the different magnetic states of CrI_3. Here we show that a change in magnetoresistance due to a switch from an antiferromagnetic to a ferromagnetic interlayer ordering in the magnetization does also exhibit a similar behavior, compatible with the FN expression of tunneling and different barrier heights in the ferro- and antiferromagnetic configurations.

To this end we generalize the original derivation by Fowler and Nordheim[9] by considering a potential barrier that even at zero bias is not spatially uniform and depends on the spin orientation. For simplicity, we consider the barrier to be constant within each layer and equal to ϕ_B^P for the spin component parallel to the magnetization of the layer

and ϕ_B^{AP} when for the spin component is antiparallel to the local magnetization. This means that in the ferromagnetic configuration at zero bias electrons with a given spin see a constant barrier height ϕ_B^P, while electrons with opposite spin feel a barrier with height $\phi_B^{AP} > \phi_B^P$. On the contrary, in the antiferromagnetic configuration, both spins experience a barrier alternating between ϕ_B^P and ϕ_B^{AP} in neighboring layers, although with opposite phase for the two spin components. At finite bias the barrier is tilted by the electric field as shown in the insets of Supplementary Figure 10, where solid and dashed lines are used to distinguish different spin components and the blue (orange) color refers to a ferromagnetic (antiferromagnetic) configuration for four layers. The tunneling current in the zero-temperature limit is computed as[10]

$$I(V) \propto \int_{E_F-eV}^{E_F} (E_F - E) \sum_\sigma T_\sigma(E)\, dE$$

where E_F is the Fermi energy (assumed to be the same in both electrodes), V is the applied bias, and $T_\sigma(E)$ is the tunneling probability for spin-σ electrons. Within the WKB approximation $T_\sigma(E)$ is given by

$$T_\sigma(E) \propto \exp\left\{-2\int_0^d \sqrt{\frac{2m^*}{\hbar^2}[V_\sigma(z) - E]}\, dz\right\}$$

where d is the overall thickness of the CrI_3 barrier. The integral is limited to regions where $V_\sigma(z) > E$, while further scattering or interference events are neglected.

In Supplementary Figure 10 we report on our results for the current in the ferromagnetic (blue) and antiferromagnetic configurations for four layers of CrI_3. We plot I/V^2 as a function of the inverse bias $1/V$ in a semi-logarithmic scale to emphasize that in both cases the current follows the FN behavior. In the ferromagnetic case, the current is not only larger in magnitude but also has a smaller FN slope with respect to the antiferromagnetic case. As mentioned in the main text, the effect can be rationalized in terms of a smaller effective barrier in the ferromagnetic case. Indeed, in this case only one spin component (parallel to the magnetization) contributes to the current with barrier height equal to ϕ_B^P. On the contrary, in the antiferromagnetic case, both spin components contribute to the current but feel a larger effective barrier in between ϕ_B^P and ϕ_B^{AP}. This is in perfect agreement with experiments if we assume that the zero-field spin configuration is antiferromagnetic, while it is ferromagnetic at larger **B**-fields. These results provide clear evidence that interpreting the magnetoresistance in terms of a change in effective barrier height is a physically reasonable point of view, which can apply also if the magnetic states are more complex than the simple ferromagnetic and antiferromagnetic interlayer configurations.

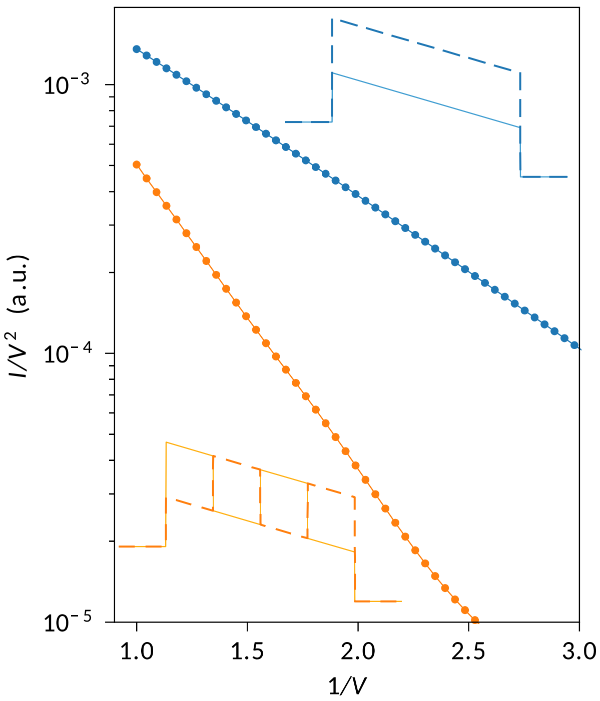

Supplementary Figure 10| Fowler-Nordheim tunneling current in the presence of a non-uniform and spin-dependent barrier. Tunneling current I computed as a function of the bias voltage V and plotted as I/V^2 versus $1/V$ in a semi-logarithmic scale to emphasize the FN behavior. The calculation has been performed considering four layers of CrI_3 with an overall thickness $d = 2.64$ nm. The barrier heights have been set to $\phi_B^P = 0.18$ eV and $\phi_B^{AP} = 0.38$ eV relative to the Fermi energy $E_F = 1.5$ eV in both electrodes at zero bias. The insets show the profile of the barrier in ferromagnetic (top, blue) and antiferromagnetic configuration (bottom, orange), where solid and dashed lines distinguish opposite spin components. In the ferromagnetic case the tunneling current is larger in magnitude and has a smaller FN slope than in the antiferromagnetic case, signaling a smaller effective barrier height.

Supplementary References:

1. McGuire M. A., Dixit H., Cooper V. R. & Sales B. C. Coupling of Crystal Structure and Magnetism in the Layered, Ferromagnetic Insulator CrI_3. *Chem. Mater.* **27,** 612-620 (2015).

2. Dillon J. F. & Olson C. E. Magnetization Resonance and Optical Properties of Ferromagnet CrI_3. *J. Appl. Phys.* **36,** 1259-1260 (1965).

3. Dillon J. F., Kamimura H. & Remeika J. P. Magneto-optical properties of ferromagnetic chromium trihalides. *J. Phys. Chem. Solids* **27,** 1531-1549 (1966).

4. Wang L., Meric I., Huang P. Y., Gao Q., Gao Y., Tran H., Taniguchi T., Watanabe K., Campos L. M., Muller D. A., Guo J., Kim P., Hone J., Shepard K. L. & Dean C. R. One-Dimensional Electrical Contact to a Two-Dimensional Material. *Science* **342,** 614-617 (2013).

5. Zhong D., Seyler K. L., Linpeng X., Cheng R., Sivadas N., Huang B., Schmidgall E., Taniguchi T., Watanabe K., McGuire M. A., Yao W., Xiao D., Fu K.-M. C. & Xu X. Van der Waals engineering of ferromagnetic semiconductor heterostructures for spin and valleytronics. *Sci. Adv.* **3,** e1603113 (2017).

6. Giannozzi P., Baroni S., Bonini N., Calandra M., Car R., Cavazzoni C., Ceresoli D., Chiarotti G. L., Cococcioni M., Dabo I., Dal Corso A., de Gironcoli S., Fabris S., Fratesi G., Gebauer R., Gerstmann U., Gougoussis C., Kokalj A., Lazzeri M., Martin-Samos L., Marzari N., Mauri F., Mazzarello R., Paolini S., Pasquarello A., Paulatto L., Sbraccia C., Scandolo S., Sclauzero G., Seitsonen A. P., Smogunov A., Umari P. & Wentzcovitch R. M. QUANTUM ESPRESSO: a modular and open-source software project for quantum simulations of materials. *Journal of Physics-Condensed Matter* **21,** (2009).

7. Thonhauser T., Zuluaga S., Arter C. A., Berland K., Schröder E. & Hyldgaard P. Spin Signature of Nonlocal Correlation Binding in Metal-Organic Frameworks. *Phys. Rev. Lett.* **115,** 136402 (2015).

8. Sohier T., Calandra M. & Mauri F. Density functional perturbation theory for gated two-dimensional heterostructures: Theoretical developments and application to flexural phonons in graphene. *Phys. Rev. B* **96,** 075448 (2017).

9. Fowler R. H. & Nordheim L. Electron emission in intense electric fields. *Proc. R. Soc. London A* **119,** 173-181 (1928).

10. Simmons J. G. Generalized formula for the electric tunnel effect between similar electrodes separated by a thin insulating film. *J. Appl. Phys.* **34,** 1793-1803 (1963).
